# Enhanced Anti-Melanoma Activity of Nutlin-3a Delivered via Ethosomes: Targeting p53-Mediated Apoptosis in HT144 Cells

**DOI:** 10.3390/cells13201678

**Published:** 2024-10-11

**Authors:** Arianna Romani, Giada Lodi, Fabio Casciano, Arianna Gonelli, Paola Secchiero, Giorgio Zauli, Olga Bortolini, Giuseppe Valacchi, Daniele Ragno, Agnese Bondi, Mascia Benedusi, Elisabetta Esposito, Rebecca Voltan

**Affiliations:** 1 Department of Translational Medicine and LTTA Centre, University of Ferrara, 44121 Ferrara, Italy; arianna.romani@unife.it (A.R.); paola.secchiero@unife.it (P.S.); 2 Department of Environmental and Prevention Sciences and LTTA Centre, University of Ferrara, 44121 Ferrara, Italy; giada.lodi@unife.it (G.L.); fabio.casciano@unife.it (F.C.); 3 Department of Environmental and Prevention Sciences, University of Ferrara, 44121 Ferrara, Italy; arianna.gonelli@unife.it (A.G.); olga.bortolini@unife.it (O.B.); giuseppe.valacchi@unife.it (G.V.); 4 Research Department, King Khaled Eye Specialistic Hospital, Riyadh 12329-8139, Saudi Arabia; gzauli@kkesh.med.sa; 5 Plants for Human Health Institute, Animal Sciences Department, NC Research Campus, NC State University, Kannapolis, NC 28081, USA; 6 Department of Food and Nutrition, Kyung Hee University, Seoul 02447, Republic of Korea; 7 Department of Chemical, Pharmaceutical and Agricultural Sciences, University of Ferrara, 44121 Ferrara, Italy; daniele.ragno@unife.it (D.R.); agnese.bondi@unife.it (A.B.); 8 Department of Neuroscience and Rehabilitation, University of Ferrara, 44121 Ferrara, Italy; mascia.benedusi@unife.it

**Keywords:** nanodelivery, ethosomes, nutlin-3a, MDM2 inhibitors, cell cycle, p53, Notch-1, melanoma

## Abstract

This study evaluated ethosomes as a novel nanodelivery system for nutlin-3a, a known MDM2 inhibitor and activator of the p53 pathway, to improve nutlin-3a’s poor solubility, limiting its bio-distribution and therapeutic efficacy. The potential of nutlin-3a-loaded ethosomes was investigated on two in vitro models of melanoma: the HT144 cell line p53^wild-type^ and the SK-MEL-28 cell line p53^mutated^. Nutlin-3a-loaded ethosomes were characterized for their physicochemical properties and used to treat melanoma cells at different concentrations, considering nutlin-3a solution and empty ethosomes as controls. The biological effects on cells were evaluated 24 and 48 h after treatment by analyzing the cell morphology and viability, cell cycle, and apoptosis rate using flow cytometry and the p53 pathway’s activation via Western blotting. The results indicate that ethosomes are delivery systems able to maintain nutlin-3a’s functionality and specific biological action, as evidenced by the molecular activation of the p53 pathway and the biological events leading to cell cycle block and apoptosis in p53^wild-type^ cells. Nutlin-3a-loaded ethosomes induced morphological changes in the HT144 cell line, with evident apoptotic cells and a reduction in the number of viable cells of over 80%. Furthermore, nutlin-3a-loaded ethosomes successfully modulated two p53-regulated proteins involved in survival/apoptosis, with up to a 2.5-fold increase in membrane TRAIL-R2 and up to an 8.2-fold decrease in Notch-1 (Notch intracellular domain, NICD) protein expression. The expression of these molecules is known to be altered or dysfunctional in a large percentage of melanoma tumors. Notably, ethosomes, regardless of their nutlin-3a loading, exhibited the ability to reduce HT144 melanoma cellular migration, as assessed in real time using xCELLigence, likely due to the modification of lipid rafts, suggesting their potential antimetastatic properties. Overall, nutlin-3a delivery using ethosomes appears to be a significantly effective means for upregulating the p53 pathway and downregulating active Notch-1, while also taking advantage of their unexpected ability to reduce cellular migration. The findings of this study could pave the way for the development of specific nutlin-3a-loaded ethosome-based medicinal products for cutaneous use.

## 1. Introduction

Nutlin-3 is a potent double minute murine 2 (MDM2) inhibitor that binds to the p53 pocket of MDM2, preventing p53 degradation and promoting its stabilization [1]. The interaction of nutlin-3 with MDM2 increases the half-life of p53 in treated tumor cells and promotes the nongenotoxic activation of p53 pathways related to cell cycle blockade and apoptosis induction. These effects, supported by the evidence that nutlin-3 does not induce apoptosis in normal healthy cells, have prompted research into the use of this molecule against several types of cancer, from solid tumors to hematological malignancies, with promising preclinical results [2,3,4]. However, the clinical use of nutlin-3 has been restricted due to its poor water solubility, limiting its biodistribution to target tissues and hampering the therapeutic effect [5,6]. For this reason, to administer nutlin-3, specialized delivery systems are needed to load the drug in a physiological aqueous dispersion, improving its bioavailability.

Cutaneous melanoma is a challenging skin tumor, correlated to more than 80% of skin-cancer-related deaths [7]. This disease has high metastatic potential due to its great ability to escape from the immune system [8]. Fortunately, 90% of cases of melanoma diagnosed early does not display any evidence of metastasis. Therapies are limited, mainly restricted to surgery combined with a cycle of radiotherapy and chemotherapy. Poor outcomes are high due to melanoma’s resistance to apoptosis, patients’ comorbidities, and toxicities [9]. The development of new effective therapeutic interventions with limited side effects may act as an adjuvant/neoadjuvant tool to fight this disease.

Skin pathologies can be treated via the topical administration of conventional semi-solid forms, such as gels and creams, or through specialized colloidal delivery systems (e.g., solid lipid nanoparticles, nanoemulsions, liposomes, and ethosomes) [10]. These new specialized delivery systems allow the loading of drugs with different physicochemical properties. Moreover, it is possible to achieve drug permeation through the skin more effectively with respect to conventional formulations, offering the chance to overcome the stratum corneum barrier that hampers drug passage. Among the specialized colloidal delivery systems, ethosomes are characterized by phosphatidylcholine bilayer nanovesicles dispersed in a hydroethanolic phase, with ethanol in the range of 20–40%, *v*/*v* [11]. Ethosomes are considered an upgrade or second generation of liposomes, possessing some advantageous characteristics, such as biocompatibility, controlled release, and the protection of carried molecules from degradation and clearance. Indeed, ethosomes’ vesicle structure is composed of lipids, similar to those constituting the stratum corneum, that allow passage through the skin. Delivery of the loaded drug into the deeper skin strata can proceed via fusion with stratum corneum lipids or via the unaltered crossing of the epithelium [12]. In addition, ethanol improves ethosomes’ elasticity and enhances the vesicles’ transdermal penetration, disorganizing the stratum corneum structure [13]. These features make ethosomes suitable for treating skin diseases, including skin cancer [11,14,15] and psoriasis [16,17]. The effectiveness of ethosomes compared to liposomes in delivering drugs for the treatment of pain and inflammation is well established. In 2019, Abd El-Alim et al. investigated the permeation and flux of diflunisal ethosomes across the skin through in vitro and in vivo experiments [18]. Their results showed that fluorescent ethosomes penetrate the skin layers more effectively than liposomes, as demonstrated by confocal laser scanning microscopy. Furthermore, a preliminary study demonstrated that quercetin induced significant effects on human keratinocytes and melanoma cells when delivered with ethosomes [19].

These features led us to design and evaluate ethosomes for nutlin-3 delivery against malignant melanoma, one of the most aggressive cancers worldwide [20,21]. Recently, our group showed that ethosomes loaded with nutlin-3a, the pure active enantiomer of nutlin-3, administered to ex vivo skin explants were able to induce protection against skin damage induced by UV radiation [22].

Taking into consideration the above evidence, the primary objective of this study was to investigate the potential application of nutlin-3a-loaded ethosomes on melanoma cell lines as a proof-of-concept tumor model.

The findings of this study may open new avenues of research involving the development of specific ethosome-based medicinal products for cutaneous use.

## 2. Materials and Methods

### 2.1. Ethosomes Preparation and Characterization

Ethosome dispersions were prepared according to a previously established protocol with slight modifications [22]. To prepare drug-loaded ethosomes, nutlin-3a ((-)-4-(4,5-bis(4-chlorophenyl)-2-(2-isopropoxy-4-methoxyphenyl)-4,5-dihydro-1H-imidazole-1-carbonyl) piperazin-2-one, 98% ee) was previously solubilized in a phosphatidylcholine ethanol solution before dropping water into it.

#### 2.1.1. Evaluation of Size Distribution and Morphology of Ethosomes

To evaluate the size distribution of ethosome vesicles, photon correlation spectroscopy (PCS) was employed by a Zetasizer Nano-S90 (Malvern Instr., Malvern, England) equipped with a 5 mW helium neon laser and a wavelength output of 633 nm. The analyses were carried out at 25 °C, at a 90° angle and with a 180 s run time. Size distributions were obtained by means of the “CONTIN” method [23] on ethosome samples diluted with bi-distilled water at a 1:20 *v*/*v* ratio. Z average mean diameters ± standard deviation (SD) were considered.

The morphology of ethosome vesicles was investigated using cryogenic transmission electron microscopy with a Zeiss/Leo EM922 Omega EFTEM Microscopy (Zeiss Microscopy GmbH, Jena, Germany), following a previously reported method [24]. The specimens were examined with reduced doses ≈1000–2000 e/nm^2^ at 200 kV, maintaining the samples at a temperature <100 K. Zero-loss filtered images (ΔE = 0 eV) were recorded by a CCD digital camera (Ultrascan 1000, Gatan, Munich, Germany), while the GMS 1.9 software (Gatan, Munich, Germany) was used for image analysis.

#### 2.1.2. Evaluation of Nutlin-3a Entrapment Capacity in Ethosomes

To quantify the amount of nutlin-3a associated with ethosomes, ultrafiltration and HPLC were employed, as previously reported [22]. Specifically, the day after preparation, a sample of the ethosome dispersion was subjected to ultrafiltration (Microcon centrifugal filter unit YM-10 membrane, NMWCO 10 kDa, Sigma-Aldrich, Darmstadt, Germany), and the retentate was diluted with ethanol, filtered (nylon membrane, 0.22 μm pore diameter), and analyzed via HPLC [24]. Briefly, a Perkin Elmer Series 200 HPLC system supplied with a micro-pump, an auto sampler, and a UV detector operating at 255 nm was employed in conjunction with a stainless-steel C-18 reverse-phase column (15 × 0.46 cm) packed with 5 μm particles (Hypersil BDS C18 Thermo Fisher Scientific S.p.A., Milan, Italy). The column was eluted with acetonitrile/water 40:60 *v*/*v*, pH 3, as a mobile phase (1 mL/min flow rate). The sample injection volume was 5 μL, while the retention time was 9 min.

The entrapment capacity (EC) was calculated as a percentage, considering the amount of nutlin-3a retained by the ethosomes with respect to the total content of the drug employed for the vesicle preparation.

### 2.2. Cells Cultures and Treatments

Human skin malignant melanoma HT144 cells (ATCC HTB-63) and A375 (ATCC CRL-1619), both expressing p53^wild-type^, and SK-MEL-28 cells (ATCC HTB-72), expressing a p53 not-active mutant, were acquired from American Type Culture Collection (ATCC, LGC, Sesto San Giovanni, Italy) and cultured in Dulbecco’s modified Eagle’s medium (DMEM, Carlo Erba, Cornaredo, Italy) supplemented with 10% fetal bovine serum (FBS, Gibco, Invitrogen Corporation, NY, USA) and 1% penicillin–streptomycin–glutamine 100X (Sigma-Aldrich, Merck KGaA, Darmstadt, Germania). In some experiments, human keratinocyte HaCat cells (AddexBio, San Diego, CA, USA were used and cultured as described above. The cells were maintained at 37 °C in a humidified atmosphere with 5% CO_2_ and were detached using 0.25% trypsin-EDTA every 2–3 days. Cultures were routinely checked for mycoplasma contamination (REP-MYS-50, Invitrogen, Waltham, MA, USA) and used below passage 20.

For experiments, cells were seeded at a density of 1.5 × 10^5^ cells/well in 6-well plates. The following day, cells were treated with several concentrations of nutlin-3a-loaded ethosomes (to final predetermined concentrations of 0.1, 1, 2.5, 5, and 10 µM of nutlin-3a) and with nutlin-3a solubilized in a 30% water/ethanol solution at the same concentrations (0.1, 1, 2.5, 5, and 10 µM). Cells grown with a complete medium (untreated) or treated with the vehicle or empty ethosomes were used as internal negative controls. The vehicle was prepared with 30% water/ethanol to replicate the ethanol concentration in the nutlin-3a solution. Ethosomes free of drugs (empty ethosomes) were used as a control with respect to nutlin-3a-loaded ethosomes.

The final ethanol volumes were inferior to 0.5% *v*/*v*. 24 and 48 h after treatment, cells were harvested for viability, cell cycle, apoptosis, Western blotting, and cell surface analyses. For the migration experiments, cell seeding and treatments are described in the next paragraphs.

### 2.3. Cell Viability, Cell Cycle Profile, and Apoptosis Assays

Cells treated for 24 or 48 h with nutlin-3a in solution, nutlin-3a-loaded ethosomes with an identical nutlin-3a concentration, and the vehicle and empty ethosomes as controls were analyzed for cell viability using Trypan blue exclusion dye (Gibco). Contextually, images of cultures were acquired by an EVOS XL microscope system (Advanced Microscopy Group, Washington, USA) for morphological analysis.

At the same time points, the cell cycle profile was examined via 5-bromodeoxyuridine (BrdU; Sigma-Aldrich) incorporation. Briefly, cells were incubated with 10 µM BrdU for 1 h at 37 °C; after incubation, floating cells and cells detached due to trypsinization were pooled, fixed with 70% ethanol, and stored at 4 °C until staining. Cells were stained with anti-BrdU primary antibody (BD Pharmingen TM, San Diego, CA, USA) for 1 h and then washed and stained with goat F (ab’)2 anti-mouse IgG (H + L) fluorescein isothiocyanate-conjugated secondary antibody (Beckman Coulter, Brea, CA, USA) and propidium iodide (PI, Sigma-Aldrich) for 1 h. Cells were then acquired using the FACS Calibur flow cytometer (BD Bioscience, San Josè, CA, USA), and the data were analyzed using FlowJo software version 9.9.6 (Tree Star, Ashland, OR, USA).

Twenty-four and forty-eight hours after treatment, cells were collected with trypsin-EDTA and pooled with floating cells to analyze the apoptosis in the entire cell population. The percentage of apoptotic cells was quantified via flow cytometry (FACS CantoII; BD Biosciences), immediately following Annexin V-FITC/PI staining (Beckman Coulter), as previously described [25]. Apoptosis data analysis was performed using the FlowJo software version 9.9.6 (Treestar, Ashland, OR, USA).

### 2.4. Western Blotting

Twenty-four h after treatment with nutlin-3a in solution, nutlin-3a-loaded ethosomes with an identical nutlin-3a concentration, and the vehicle and empty ethosomes as controls, cells were harvested for protein expression analysis via Western blotting. Cells were lysed in a buffer containing 50 mM Tris-Cl, with a pH of 7.5, 150 mM of NaCl, 0.1% sodium dodecyl phosphate, 1% NP40, 0.25% sodium deossicolate, and a protease and phosphatase inhibitor cocktail (Thermo Fisher, Waltham, MA, USA). The protein concentration of extracts was determined by means of the bicinchoninic acid (BCA) Protein Assay Kit (Pierce™, Thermo Fisher Scientific, MA, USA). For Western blotting analysis, equal amounts of protein were separated on 10% and 12.5% SDS-PAGE gels and transferred onto nitrocellulose membranes (Amersham, Merk, Darmstadt, Germany). Each gel was loaded with a specific molecular weight marker (1610374 Biorad, Hercules, CA, USA).

The membranes were incubated with the following antibodies: anti-p53 (clone DO-1), anti-Mdm2 (clone SMP14), (all from Santa Cruz Biotechnology, Santa Cruz, CA, USA), anti-p21 (polyclonal rabbit anti-human, cat. numb. 10355-1AP, Protein Tech, Manchester, UK), Notch-1 cleaved (clone D3B8, Notch-1 intracellular domain, NICD, Cell Signaling Technology, Danvers, MA, USA), and anti-tubulin (clone TUB 2.1, Sigma-Aldrich). After incubation with secondary antibody (polyclonal goat anti-mouse IgG peroxidase-conjugated cat. numb. A4416; or polyclonal goat anti-rabbit IgG peroxidase-conjugated, cat. numb. A6254, both from Sigma-Aldrich), immunoreactive protein bands were detected with the enhanced chemiluminescent (ECL) Lightning kit (Advansta Inc., San Jose, CA, USA). The acquisition was performed using an Image QuantTM LASS 4000 imager (GE Healthcare Biosciences, Chalfont Saint Giles, Buckinghamshire, United Kingdom). Densitometric values were estimated using the Fiji software version 9.3.1 [26], with slight brightness and contrast adjustments to the entire image. Protein levels were first normalized to the tubulin expression as a loading control, and the following ratio vs. the untreated value was calculated for each experiment. The relative densities of at least three independent experiments were used for statistical analyses.

### 2.5. Cellular Surface Analysis

The surface expression of TRAIL-R2 (tumor necrosis factor-related apoptosis-inducing ligand-receptor 2) was evaluated using flow cytometric analysis. Cells were treated for 48 h with nutlin-3a in solution, nutlin-3a-loaded ethosomes, and the vehicle and controls as described above, harvested, and then stained with anti-human PE-conjugated antibody against TRAIL-R2 (clone 71908, R&D Systems, Minneapolis, MN, USA). A mouse IgG_2B_ PE-conjugated antibody (clone 133303, R&D Systems) was used to determine the level of nonspecific staining. The LIVE/DEAD^TM^ fixable far red dead cell stain (eBioscience, San Diego, CA, USA) was added to the staining mix to exclude dead cells. Data collection was performed on a FACS CantoII instrument using BD FACS Diva software (all from BD Biosciences), and the median fluorescence values were analyzed using FlowJo software version 9.9.6 (Treestar).

### 2.6. Real-Time Cell Migration

Experiments were performed using a DP-RTCA xCELLigence real-time cell analyzer (Agilent, Santa Clara, CA, USA), which records changes in impedance as the Cell Index (CI) using electronically integrated Boyden chambers.

Before seeding, cells were starved for 2 h with DMEM containing 0.1% FBS, 1% pen/strep, and L-glut (DMEM 0.1%). Cells were then detached with trypsin, seeded (40,000 cells/well) in the upper chamber of CIM plates (Agilent) in DMEM 0.1%, and exposed to nutlin-3a, nutlin-3a-loaded ethosomes, and controls. The kinetics of migration through the lower chamber, filled with DMEM containing 5% FBS as a chemoattractant, were assessed in duplicate and CI was recorded continuously every 5 min for 24 h using the RTCA software (version 2.8.1, Agilent). Negative migration controls were established by using DMEM 0.1% in the lower chamber. The areas under the curves, in the range 1–15 h, were analyzed by GraphPad Prism version 8 software (GraphPad Software, San Diego, La Jolla, CA, USA) and expressed as a percentage of migration with respect to the untreated cultured set. Analyses were performed on four independent experiments.

### 2.7. Statistical Analyses

Statistical analysis of data was performed using GraphPad Prism version 8 software (GraphPad Software). Data obtained from at least three independent experiments were tested for normal distribution using the Shapiro–Wilks test. The results were evaluated through analysis of variance (ANOVA), followed by Bonferroni’s post hoc test for multiple corrections. The results are expressed as the mean ± standard error of the mean (SEM), and significance was defined as *p* < 0.05.

## 3. Results

### 3.1. Ethosomes

To solubilize nutlin-3a in a physiological delivery system suitable for cutaneous administration, ethosomes were prepared and characterized. The insolubility of nutlin-3a in water requires specialized delivery systems. Specifically, due to the drug’s slight solubility in ethanol (5.69 mg/mL), we chose to employ ethosomes, aqueous colloidal dispersions of phosphatidylcholine containing ethanol at a high percentage, with the formulation reported in Table 1 in our previous study [22].

The simple dropping of water into ethanolic solutions of phosphatidylcholine under stirring resulted in milky homogeneous dispersions. As reported in Table 1, the Z average mean diameters determined by PCS were 212 nm for ethosomes and 224 nm for nutlin-3a-loaded ethosomes, suggesting that the drug’s presence slightly affected the vesicle size, as shown in a recent study by our group [22]. In addition, polydispersity indexes indicated homogeneous size distributions, being 0.12 and 0.17 in the case of empty and drug-loaded ethosomes, respectively [23]. The polydispersity index is a critical quality parameter, indicating the degree of homogeneity of particles or vesicles, and its value can range from 0 to 1, with lower values corresponding to a more monodisperse size distribution. The observed polydispersity index values, at less than 0.2, suggest an uniform size distribution and physical stability of the vesicular dispersions [27]. Indeed, the physical stability of ethosomes can be affected by several factors, including the size distribution of the lipid vesicles. In cases of high polydispersity, larger vesicles are prone to aggregation and sedimentation. Moreover, the monodispersity of nanodelivery systems is crucial since the size affects particles’/vesicles’ interactions with the body districts [28].

The CONTIN method was employed to determine size distribution by PCS, being an effective and reliable way to obtain the size distribution of colloidal suspensions. Alternatively, in the case of suspensions with sharp size distributions, the monomodal cumulant analysis can be employed to determine the relative width of the size distribution. We preferred to employ the CONTIN method that does not require a priori assumption of a certain type of distribution [29].

**Figure 1:**
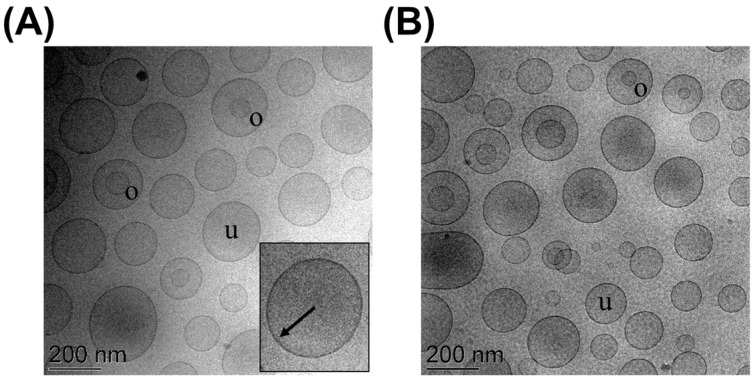
Cryo-TEM images of unloaded (**A**) and nutlin-3a-loaded (**B**) ethosomes. o: oligolamellar vesicles; u: unilamellar vesicles. The bar equals 200 nm in both panels. The arrow in the inset of panel A indicates the phosphatidylcholine double layer.

The vesicles’ morphology, shown in the cryo-TEM images reported in Figure 1, revealed unilamellar (u) and oligolamellar (o) spherical vesicles, both in the case of unloaded (Figure 1A) and nutlin-3a-loaded (Figure 1B) ethosomes. In the inset of panel A, the bilayer of a magnified ethosome is shown more clearly.

Notably, the ethosomes’ morphology does not change in the absence or in the presence of nutlin-3a. The EC of nutlin-3a calculated after ultrafiltration and HPLC analysis was around 90% (Table 1), confirming the suitability of ethosomes as vehicles for nutlin-3a loading. Indeed, ethosomes contain 90% of the nutlin-3a within the disperse phase, consisting of vesicles, while the remaining 10% is free in the dispersing phase. Thus, the total amount of nutlin-3a employed for ethosomes’ preparation is found in ethosomes (90% in the inner and the remaining in the outer phase).

#### Validation of Nutlin-3a HPLC Method

To validate the HPLC method employed for nutlin-3a analysis, some key parameters were calculated, namely selectivity/specificity, linearity, repeatability, limit of detection (LOD), and limit of quantification (LOQ), as reported in the ICH guideline Q2(R2) on validation of analytical procedures [30]. The chromatograms did not show the presence of other substances (i.e., impurities or degradation products), demonstrating the selectivity/specificity of the analytical procedure. A linear relationship between nutlin-3a concentration and response was obtained: R^2^ = 0.9995. The repeatability, expressed as relative standard deviation, was below 0.05% (n = six injections). LOD and LOQ values were 0.053 and 0.160 μg/mL, respectively, further supporting the validity of the method.

### 3.2. Nutlin-3a’s Delivery by Ethosomes Efficiently Reduces the Viability of p53^wild-type^ Melanoma Cells

The biological effects of the nutlin-3a-loaded ethosomes on HT144 and SK-MEL-28 melanoma cell lines were evaluated as preclinical models of melanoma. HT144 cells express p53^wildtype^ and are potential responders to nutlin-3a treatment, while SK-MEL-28 cells express p53^mutated^ (not functional) are expected non-responders and thus are useful as a negative control.

**Figure 2:**
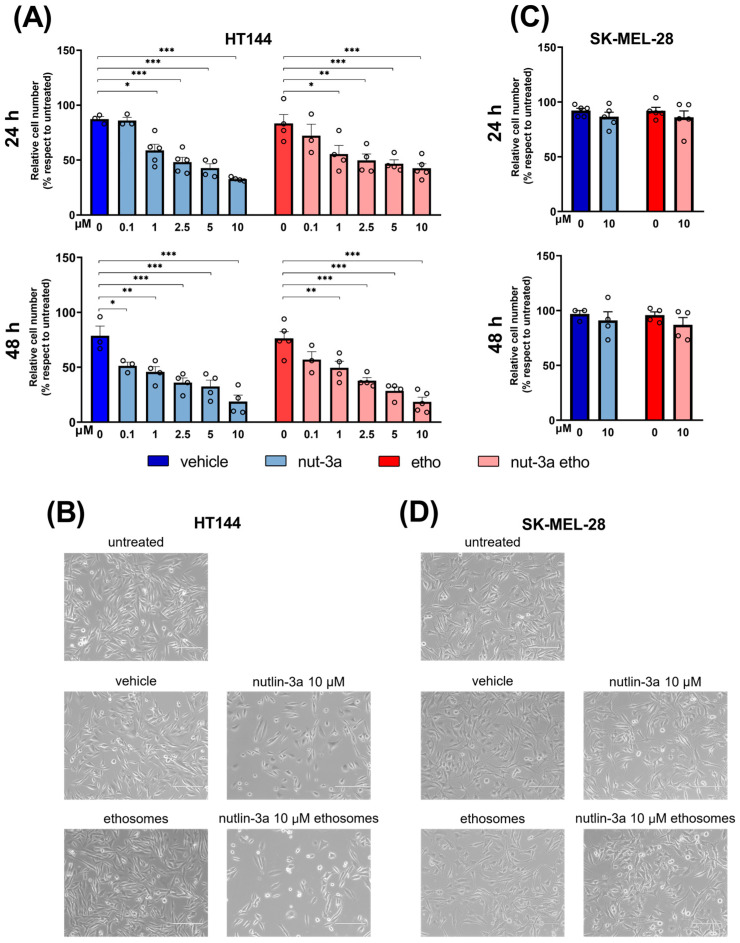
Nutlin-3a-loaded ethosomes reduce the number of viable HT144 melanoma cells. Number of viable cells evaluated via trypan blue dye exclusion of cell cultures treated with nutlin-3a or nutlin-3a-loaded ethosomes with equivalent nutlin-3a concentrations after 24 and 48 h of HT144 expressing p53^wild-type^ (**A**) and SK-MEL-28 expressing p53^mut^ (**C**). Vehicle and empty ethosomes are reported as controls. Data are calculated as percentages with respect to the untreated cells (set to 100%). Bars represent the mean ± SEM, and each circle denotes the value of each experimental replicate. Statistical analysis was performed via ANOVA followed by Bonferroni’s post hoc test. The number of asterisks indicates the relative *p*-value (*p*): *, *p* ≤ 0.05; **, *p* ≤ 0.01; ***, *p* ≤ 0.001. Representative brightfield images of cell cultures acquired with an EVOS digital microscope after 48 h of treatment are shown with a magnification of 20× (**B**,**D**).

Cell viability was assessed 24 and 48 h after treatment (Figure 2). Analyses showed that the HT144 cells’ (p53^wild-type^) viability was significantly reduced with increasing concentrations of both nutlin-3a and nutlin-3a-loaded ethosomes, with respect to the controls, reaching less than 20% after 48 h of 10µM treatment (Figure 2A), as confirmed by microscopic examination (Figure 2B).

At the same time point, morphological analyses revealed an increase in the number of floating rounded cells (i.e., apoptotic cells) compared to attached stellate cells in samples treated with nutlin-3a and nutlin-3a-loaded ethosomes. No significant difference between nutlin-3a and nutlin-3a-loaded ethosomes was observed. On the contrary, the viability and morphology of the SK-MEL-28 cell line (expressing p53^mut^) were not altered either by nutlin-3a or by nutlin-3a-loaded ethosomes (compared to controls, *p* > 0.05) (Figure 2C,D), confirming the role of p53^wild-type^ in mediating these effects. The p53-dependent activity of both nutlin-3a and nutlin-3a-loaded ethosomes was also confirmed in another melanoma cell line expressing p53^wild-type^, A374 cells (Supplementary Figure S1). Overall, treatments with the vehicle alone and empty ethosomes did not interfere with cell viability (compared to untreated cells, *p* > 0.05) or the morphology of the tested cell lines. Moreover, empty ethosomes and nutlin-3a-ethosomes, assessed at the highest concentration used in the experiments with melanoma cell lines, were not cytotoxic in HaCat human keratinocytes (Supplementary Figure S2).

### 3.3. Cytotoxicity Mediated by Nutlin-3a-Loaded Ethosomes on Melanoma Cells Is Dependent on Cell Cycle Blockade and Apoptosis Induction

To deepen the observations made regarding viability in the two cellular models, we analyzed the biological effects on cell cycle and apoptosis induction.

**Figure 3:**
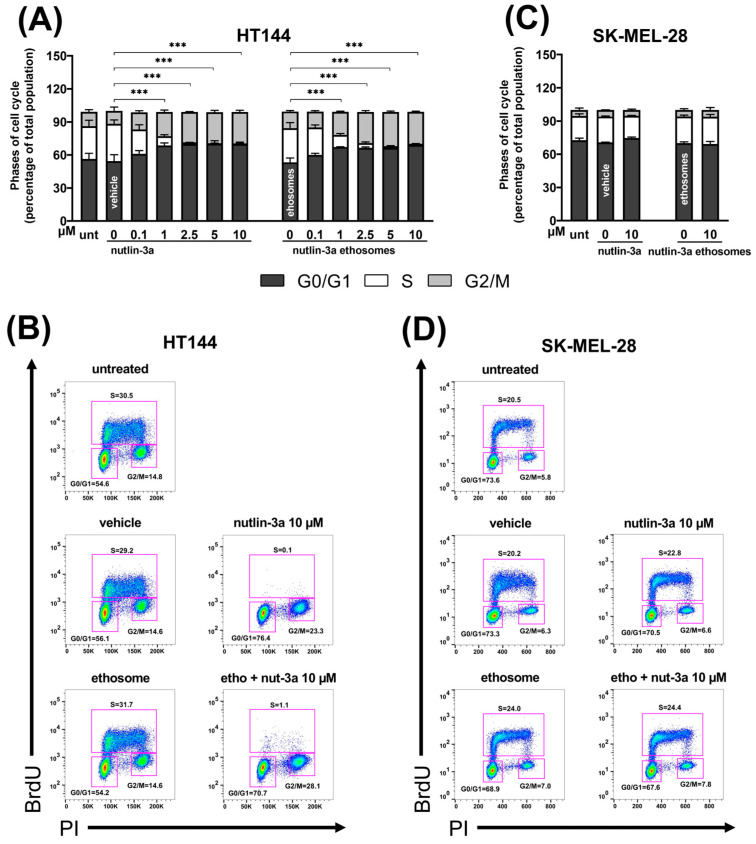
Nutlin-3a-loaded ethosomes induce cell cycle arrest in p53^wild-type^ melanoma cells. Cell distribution in the different phases of the cell cycle of HT144 expressing p53^wild-type^ (**A**) and SK-MEL-28 expressing p53^mut^ (**C**) cultures treated with nutlin-3a or nutlin-3a-loaded ethosomes with equivalent nutlin-3a concentrations after 24 h. Vehicle and empty ethosomes are reported as controls. Data are reported as the mean ± SEM from three independent experiments. Phase S statistical analysis was performed by means of ANOVA followed by Bonferroni’s post hoc test. *** indicates a *p*-value ≤ 0.001. Results are expressed as a percentage of the total population. Representative flow cytometry dot plots are shown, with rectangles framing the different cell cycle phases: S, G0/G1 and G2/M (**B**,**D**).

Cell cycle analyses, reported in Figure 3, showed a dose-dependent cell cycle block with significant S phase reduction, represented in white in panels A and C, and consequent G2/M phase accumulation, on HT144 cells treated with nutlin-3a (S phase: 1 µM, 2.5 µM, 5 µM, and 10 µM, *p* < 0.001; G2/M phase: 2.5 µM, 5 µM, and 10 µM, *p* < 0.001; G0/G1 phase: 5 µM, *p* = 0.0457, and 10 µM, *p* = 0.0157 with respect to vehicle), and nutlin-3a-loaded ethosomes (S phase: 1 µM, 2.5 µM, 5 µM, and 10 µM, *p* < 0.001; G2/M phase: 2.5 µM, 5 µM, and 10 µM, *p* < 0.001; G0/G1 phase: 10 µM, *p* = 0.0103 with respect to ethosomes alone) (Figure 3A,B). On the contrary, no alteration to cell cycle phase distribution was observed on the SK-MEL-28 cells, characterized by p53^mut^, compared to relative controls (*p* > 0.05) (Figure 3C,D).

In both cell lines, the vehicle and empty ethosomes did not interfere with the cell cycle with respect to the untreated cultures run in parallel (*p* > 0.05).

**Figure 4:**
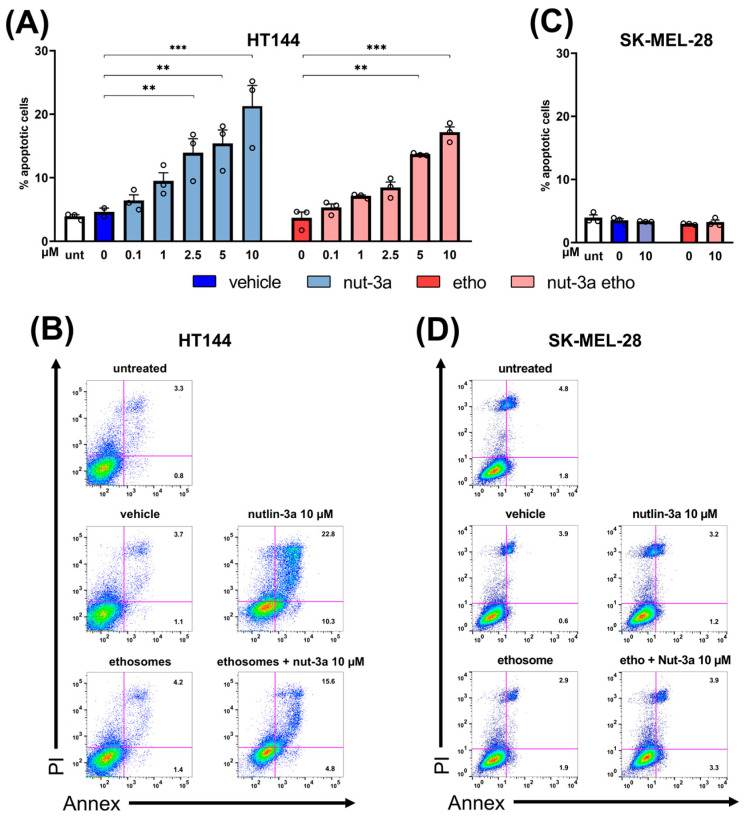
Nutlin-3a-loaded ethosomes induce apoptosis in p53^wild-type^ melanoma cells. Percentages of apoptotic cells in HT144 expressing p53^wild-type^ (**A**) and SK-MEL-28 expressing p53^mut^ (**C**) cultured with nutlin-3a or nutlin-3a-loaded ethosomes with equivalent nutlin-3a concentrations after 48 h. Vehicle and empty ethosomes are reported as controls. Results are expressed as a percentage of the total population. Bars represent the mean ± SEM, and each circle denotes the value of each experimental replicate. Statistical analysis was performed by means of ANOVA followed by Bonferroni’s post hoc test. The number of asterisks indicates the relative *p*-value (*p*): **, *p* ≤ 0.01; ***, *p* ≤ 0.001. Representative flow cytometry dot plots are shown (**B**,**D**).

Similarly, apoptosis analyses performed after annexin-PI staining showed sensibility to nutlin-3a and nutlin-3a-loaded ethosomes in HT144 cells (Figure 4A,B) and no response in SK-MEL-28 cells (Figure 4C,D). In the p53^wild-type^ cell line, a dose-dependent apoptosis induction was evident as a trend 24 h after treatment with nutlin-3a and nutlin-3a-loaded ethosomes (Supplementary Figure S3) and became significant at the 48 h’ time point (Figure 4A,B). The vehicle and empty ethosome controls were confirmed to not affect this pathway in both cell lines.

Altogether, these results indicate that the cytotoxic effects, leading to a viability reduction in HT144 cells by nutlin-3a and nutlin-3a-loaded ethosomes, are due to the joint effects of cell cycle blockage and programmed cell death induction.

### 3.4. Cytotoxicity Mediated by Nutlin-3a-Loaded Ethosomes on Melanoma Cells Is Dependent on p53 Pathway Activation

**Figure 5:**
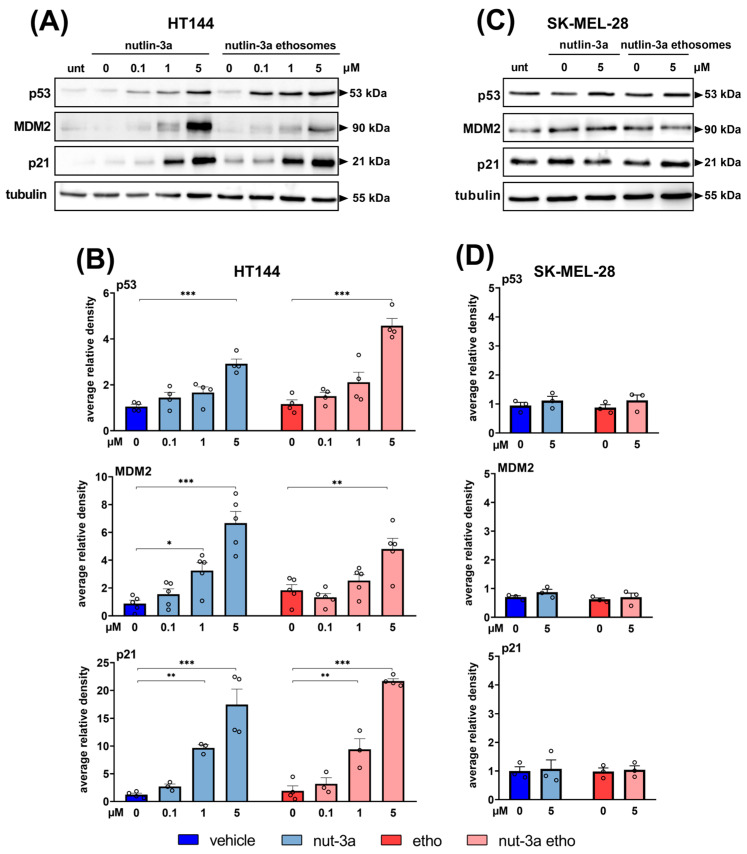
Nutlin-3a-loaded ethosomes induce p53 pathway in p53^wild-type^ melanoma cells. Representative Western blotting images of HT144 (**A**) expressing p53^wild-type^, and of SK-MEL-28 (**C**) expressing p53^mut^, treated with nutlin-3a and nutlin-3a-loaded ethosomes with equivalent nutlin-3a concentrations after 24 h. HT144 (**B**) and SK-MEL-28 (**D**). Bars represent the mean ± SEM of densitometric analyses, and each circle denotes the value of each experimental replicate. Vehicle and empty ethosomes are reported as controls. Data are normalized against tubulin, as an internal control for protein sample loading. Average relative density was calculated as a ratio with respect to untreated cells (set to 1) and reported as the mean ± SEM. Statistical analysis was performed by means of ANOVA followed by Bonferroni’s post hoc test. The number of asterisks indicates the relative *p*-value (*p*): *, *p* ≤ 0.05; **, *p* ≤ 0.01; ***, *p* ≤ 0.001.

Since both the cell cycle and apoptosis are p53-dependent events, next, we evaluated the molecular effects of the inhibition of MDM2, the specific target of nutlin-3a, and a known p53 inhibitor. After treatment for 24 h with increasing concentrations of nutlin-3a or nutlin-3a-loaded ethosomes and relative controls, we analyzed the levels of p53 and of its target genes MDM2 and p21 (Figure 5). As shown in Figure 5A,B, both nutlin-3a and nutlin-3a-loaded ethosomes significantly upregulate p53 levels in HT144 cells. The specific transcriptional activity of p53 allowed a significant upregulation of its target proteins MDM2 and p21, suggesting that the loaded drug was functional and properly internalized by the cells without loss of its biological activity. Interestingly, p53 levels were significantly higher in cells exposed to 5µM nutlin-3a-loaded ethosomes than those exposed to nutlin-3a (*p* = 0.0009). In parallel, experiments conducted on SK-MEL-28 cells expressing a functionally inactive p53 showed that the p53 pathway was not activated and the p53, MDM2, and p21 protein levels were unaffected by treatments with nutlin-3a and nutlin-3a-loaded ethosomes (Figure 5C,D). The vehicle alone and the empty ethosomes, used as controls, did not affect the p53 pathway in both cell lines.

### 3.5. Nutlin-3a-Loaded Ethosomes Induce the Surface Expression of TRAIL-R2 Receptors on p53^wild-type^ Melanoma Cells

Recent studies have shown that in melanoma cells with different sensitivities to TRAIL, cell cycle blockade correlates with an increase in sensitivity to TRAIL [31,32]. After observing that nutlin-3a and nutlin-3a-loaded ethosome treatments inhibit the cell cycle of HT144 cells and considering that the TRAIL-R2 receptor (death receptor 5, DR5) is a bona fide target of p53 [33], we evaluated the effects of treatment with nutlin-3a alone or with ethosomes on the membrane expression of TRAIL-R2.

**Figure 6:**
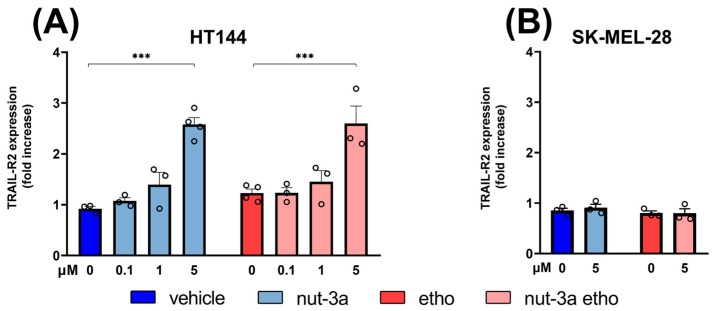
Nutlin-3a-loaded ethosomes upregulate surface death receptors TRAIL-R2 of p53^wild-type^ melanoma cells. Surface cytofluorimetric analysis of HT144 expressing p53^wild-type^ (**A**) and SK-MEL-28 expressing p53^mut^ (**B**) cell cultures treated with nutlin-3a or nutlin-3a-loaded ethosomes with equivalent nutlin-3a concentrations after 24 h. Vehicle and empty ethosomes are reported as control vehicles. Data are calculated as the fold increase in median fluorescence values with respect to untreated cells (set to 1). Bars represent the mean ± SEM, and each circle denotes the value of each experimental replicate. Statistical analysis was performed by means of ANOVA followed by Bonferroni’s post hoc test. *** indicates a *p*-value ≤ 0.001.

Surface analysis by flow cytometry showed a dose-dependent increase in TRAIL-R2 expression after treatment with nutlin-3a and nutlin-3a-loaded ethosomes in HT144 cells (expressing p53^wild-type^), with a significant increase with respect to the relative controls at a concentration of 5µM (Figure 6A). In the SK-MEL-28 cell line (expressing p53^mut^), no significant modulation of TRAIL-R2 expression was observed (Figure 6B). Interestingly, the upregulation of surface TRAIL-R2 and the apoptosis level induced by treatments are directly correlated (Spearman r = 0.85; *p* < 0.01).

### 3.6. Nutlin-3a-Loaded Ethosome Treatment Reduces Notch-1 Intracellular Domain Levels in Melanoma Cells

It has been observed that Notch-1 signaling has implications for tumor transformation and the progression and immunosuppression of melanoma [34,35,36], and that there is crosstalk between Notch-1 and p53 during cancer proliferation [37]. For these reasons, we decided to study the effects of nutlin-3a alone and nutlin-3a-loaded ethosomes on Notch-1 through the evaluation of the active Notch-1 intracellular domain (NICD) levels.

**Figure 7:**
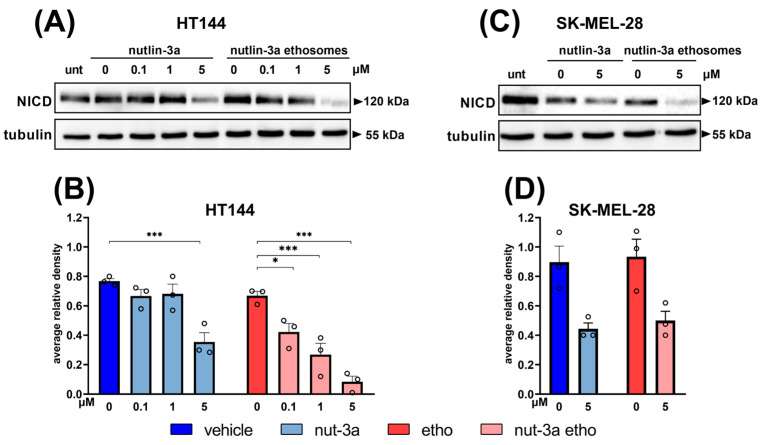
Nutlin-3a-loaded ethosomes downregulate the pro-survival active form of Notch-1 (NICD). Representative Western blotting images of HT144 (**A**), expressing p53^wild-type^, and of SK-MEL-28 (**C**), expressing p53^mut^, treated with nutlin-3a and nutlin-3a-loaded ethosomes with equivalent nutlin-3a concentrations after 48 h. HT144 (**B**) and SK-MEL-28 (**D**); densitometric analyses of three independent experiments are shown. Vehicle and empty ethosomes are reported as controls. Data are normalized against tubulin, as an internal control for protein sample loading. Average relative density was calculated as a ratio with respect to untreated cells (set to 1). Bars represent the mean ± SEM, and each circle denotes the value of each experimental replicate. Statistical analysis was performed by means of ANOVA followed by Bonferroni’s post hoc test. The number of asterisks indicates the relative *p*-value (*p*): * *p* ≤ 0.05; *** *p* ≤ 0.001.

Analyses of the Western blotting results, shown in Figure 7, indicated a significant reduction in NICD levels in HT144 cells (expressing p53^wild-type^) after 48 h of treatment with nutlin-3a and nutlin-3a-loaded ethosomes (Figure 7A,B). Interestingly, the NICD downregulation mediated by nutlin-3a-loaded ethosomes was significantly higher than that mediated by nutlin-3a (0.1µM, *p* = 0.0429; 1µM, *p* = 0.0004; 5µM, *p* = 0.0202) and initiated at a low concentration (Figure 7B). An inhibitory effect can also be observed in SK-MEL-28 cells treated with nutlin-3a and nutlin-3a-loaded ethosomes, although this modulation is not significant at the tested concentrations (Figure 7C,D).

### 3.7. Ethosomes Reduce Melanoma Cell Migration as a Delivery System-Dependent Effect

**Figure 8:**
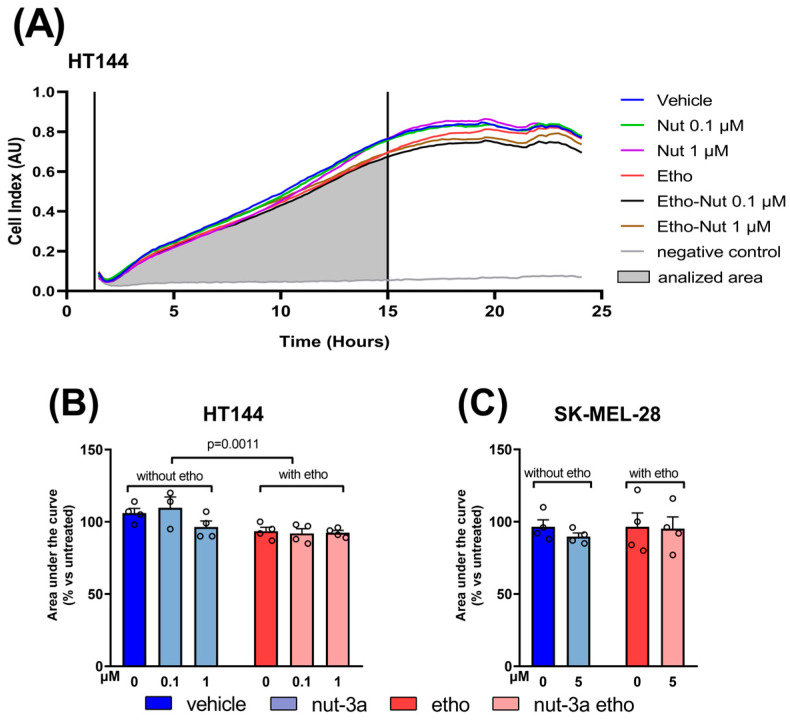
Ethosomes reduce the cell migration of HT144 cells. (**A**) Overtime Cell Index plot from RTCA software of a representative experiment. Areas under the curves in the range of 1–15 h were considered for the analysis of each experiment. Analyses of the real-time cell migration of HT144 expressing p53^wild-type^ (**B**) and SK-MEL-28 expressing p53^mutated^ (**C**) cells treated with low concentrations of nutlin-3a or nutlin-3a-loaded ethosomes with equivalent nutlin-3a concentrations. Vehicle and empty ethosomes are reported as controls. Cells were seeded into the upper chambers of the CIM plate and treated as described. Real-time monitoring of migration through the lower chambers was performed with the xCELLigence system (DP-RTCA) for 24 h. The chemoattractant for migration was FBS in the medium: 0.1% FBS in the upper chambers and 5% FBS in the lower chambers. Negative controls were performed by using 0.1% FBS medium in both chambers. Data are expressed as a percentage with respect to untreated cells (set to 100%). Bars represent the mean ± SEM, and each circle denotes the value of each replicate experiment (**B**,**C**). Statistical analysis was performed by means of a two-way ANOVA. The *p*-value of *p* = 0.0011 indicates a significant difference when comparing the groups “without ethosomes” and “with ethosomes”.

Considering the high impact of metastasis in the clinical progression of melanoma, we evaluated the potential impact of nutlin-3a and nutlin-3a-loaded ethosomes in cellular migration by using real-time quantitative impedance analysis with an xCELLigence RTCA DP system (Figure 8). To avoid apoptosis’ interference on HT144 cell cultures, experiments were set up at early time points using low concentrations of nutlin-3a and nutlin-3a-loaded ethosomes.

Unexpectedly, the analysis of migratory data, continuously collected until 15 h of treatment, brought to light significant migration inhibition by ethosomes on HT144 cell cultures (Figure 8A,B), while the effect of the drug nutlin-3a (naked or loaded in ethosomes) seemed uninfluential in limiting cell migration (two-way ANOVA; I factor: the presence of ethosomes, DF = 1, F = 15.69, *p* = 0.0011; II factor: nutlin-3a concentration, DF = 2, F = 2.033, *p* = 0.1635; interaction of two factors: DF = 2; F = 1.928, *p* = 0.1778). Meanwhile, no significant effects were observed in the SK-MEL-28 cell line (Figure 8C).

## 4. Discussion

The skin is a well-organized organ that, with its occluding and tight junctions, lipids, and keratin, serves as the body’s first line of defense [12,38]. However, this protective barrier can make the topical treatment of skin pathologies challenging [5,6]. To overcome this, various technological approaches and delivery systems are employed to facilitate drug penetration [11,12,13,14,15,16].

In this light, this study proposes ethosomes loaded with nutlin-3a for the treatment of melanoma. These ethosomes are composed of phosphatidylcholine vesicles dispersed in water. The lipid used for ethosome preparation is soy phosphatidylcholine, a glycerophospholipid widely used in colloidal system production. These phospholipids exhibit self-aggregation in water, form liquid crystalline structures, have a strong affinity for stratum corneum lipids, and are highly biocompatible [39]. Notably, the phospholipid’s nature can influence the vesicles’ stability, drug loading, as well as skin penetration. Indeed, some studies have employed positively or negatively charged phospholipids (e.g., 1,2-dioleoyl-3-trimethylammonium-propane [chloride salt], DOTAP, or 1,2-dipalmitoyl-sn-glycero-3-phosphatidylglycerol, DPPG) to improve these properties [18]. In the present research, we chose to employ an uncharged phospholipid due to its established safety and biocompatibility [40]. Absence of cytotoxicity was already demonstrated in our previous work, where we reported the use of ethosomes and nutlin-3a-loaded ethosomes on human skin explants [22].

Overall, our results show that nutlin-3a can be combined with ethosomes, maintaining molecule stability, and that nutlin-3a-loaded ethosomes can be administered to cells without loss of functionality and specific biological action. Indeed, p53^wild-type^ melanoma cells responded to the delivered drug with the molecular activation of the p53 pathway and biological events leading to cell cycle block and apoptosis, while p53^mutated^ melanoma cells displayed resistance to the treatments. These observations also demonstrate the interesting, and not at all guaranteed, anti-tumoral effects of nutlin-3a in the melanoma models used for the investigations. Previous works published in the literature have investigated the efficacy of nutlin-3 in other skin melanoma models, predominantly human A375 and mouse B16 cells as p53^wild-type^ models and human CHL-1 cells as a p53^mutated^ model [41,42,43].

Interestingly, we observed that the levels of MDM2 after treatment with nutlin-3a ethosomes are lower, and that the levels of p53 are coherently higher, compared to those measured after treatment with nutlin-3a alone (at 5 µM concentration) in HT144 p53^wild-type^ cells. This phenomenon could be related to a difference in p53–MDM2 feedback-loop oscillations due to the “stress” induced by ethosomes. Indeed, some mathematical models have been used to study this regulatory mechanism and the oscillatory waves of the two proteins, also in response to nutlin-3 [44,45]. Accordingly, we can hypothesize that ethosomes might induce a modification in the waves of these natural or drug-induced oscillations. On the other hand, a further explanation could be found with the enhanced auto-degradation caused by self-ubiquitination and/or enhanced proteasome-mediated degradation of the MDM2 protein. One possible mechanism might depend on the PKB/Akt axis, since PKB/Akt has an association with the membrane through lipid interactions [46]. If membrane lipids change in composition or fluidity (such as when ethosomes reach the cell membrane), PKB/Akt binding to the membrane could be modified and its activation reduced. One of PKB/Akt pleiotropic functions is to control the p53–MDM2 feedback loop via the phosphorylation of MDM2 [47]. From this perspective, if the PKB/Akt axis is less active, MDM2 is less phosphorylated and more susceptible to degradation. All these theories are speculations that could be investigated to better understand the role of ethosomes as pharmacological adjuvants and not only as a neutral delivery system.

Additionally, we also observed that nutlin-3a enhanced the surface expression of TRAIL-R2. This feature could sensitize p53^wild-type^ melanoma cells to TRAIL treatment and help in addressing TRAIL resistance. Indeed, it has been demonstrated by several authors that the redistribution of TRAIL receptors in membrane rafts of tumoral cells may facilitate TRAIL signal transduction and cell death [48,49]. Redistribution can be promoted by oxidative stress, as induced by pharmacological treatments like resveratrol, ceruloplasmin, and nutlin-3 [48,49,50].

Remarkably, in previous studies, we demonstrated that ethosomes can penetrate various cell types [24,51,52]. Combined fluorescence microscopy and transmission electron microscopy analyses unequivocally demonstrated the uptake of ethosomes, confirming their uptake by keratinocytes, fibroblasts, and myoblasts. Specifically, ethosomes were found in the cytoplasm, while also occurring close to the plasma membrane but never inside endosomes. In this respect, the cellular uptake does not seem to take place via classical endocytic processes, since no plasma membrane invagination typical of early endocytosis was found [52]. Ethosomes possess chemical and structural affinity with the plasma membrane; indeed, phosphatidylcholine represents a major phospholipid component of biological membranes, suggesting a possible fusion process between ethosomes and cell membranes. This phenomenon could be further promoted by the presence of ethanol, which can induce the loosening of lipid packaging in the plasmalemma region in contact with the vesicles.

Interestingly, nutlin-3a delivery by ethosomes was advantageous in HT144 cells, enhancing the downregulation of active Notch-1 with respect to soluble nutlin-3a. To discuss this result, some considerations should be made about the chemical composition of ethosomes. The supramolecular structure of ethosome vesicles, in which phosphatidylcholine is organized in bilayers, is similar to that of cell membranes. The affinity between ethosomes and cell membranes could promote a surface interaction, possibly modifying the delicate asymmetry of membrane lipid distribution. Integral membrane proteins respond to the asymmetric distribution of lipids and can modify their properties, causing alterations in cell physiology [53]. Several integral membrane proteins are involved in lipid movements, including flippases, floppases, and scramblases [53,54]. Scramblases have been shown to facilitate the exposure of phosphatidylserine, which serves as a signal molecule for the activation of disintegrin and metalloproteinase proteases (ADAMs) ADAM10 and ADAM17 [54,55]. Following this activation and the change in membrane fluidity, ADAM10/17 approaches its substrate and cuts through it, activating various extra- and intracellular signals. ADAM promotes the cleavage of Notch-1 extracellular domain (NECD) from the transmembrane Notch-1 intracellular domain (TM-NICD) (S2 cleavage), while γ-secretase promotes the release of NICD from the TM domain (S3 cleavage). Regarding the observed downregulation of NICD in our model, we can hypothesize that the activity of the γ-secretase in lipid rafts is altered [56]. Such cell membrane microdomains could be changed by ethosome uptake, and the altered balance of membrane lipids and/or the modification of their fluidity could modify the activity of this enzyme.

Given the complex reciprocal relationship between p53 and Notch-1, as described in some tumoral models [37], it was quite surprising that the effect of nutlin-3a treatment could also modulate NICD levels in the cell line expressing mutated p53. However, it has been shown that MDM2 can activate, and not degrade, Notch-1 via ubiquitination in several cellular models [57,58]. For this reason, it can be hypothesized that MDM2 inhibition interfered with Notch-1 activation in SK-MEL-28 as an off-target effect of nutlin-3a. Despite this, however, the reduction in NICD levels had a minimal effect on cell proliferation and apoptosis, and the cell line displayed resistance to nutlin-3a treatment.

Remarkably, the ethosomes reduced melanoma cell migration of the HT144 cell line, suggesting a possible anti-metastatic role driven by the delivery system since nutlin-3a has not effect on migration in the investigated models. One of the hypotheses explaining this result involves the potential role of lipid rafts, since they are involved in the processes of cell adhesion and migration and tumor metastasis [59,60]. Supporting this thesis, previous studies reported that the alteration of the lipid rafts’ composition accounted for the efficacy of liposome-encapsulated doxorubicin on colon cancer cells [61] and that lipid rafts reorganization after the treatment of the cancer cells with the raft-targeted drug, edelfonsine, had potent anti-metastatic effects [62]. Another work demonstrated that the disruption of lipid rafts inhibited the metastasis of cancer cells by reducing lamellipodia formation [63].

Recently, our research group demonstrated the skin penetration capacity of nutlin-3a-loaded ethosomes in an ex vivo skin explant [22]. Transmission electron microscopy of skin samples, mounted in a bioreactor and treated with ethosomes for 1 and 3 h, demonstrated that vesicles were able to enter the skin, while maintaining the vesicle structure. Indeed, intact ethosomes were found in keratinocytes and even in the dermis after 3 h of incubation. Based on these remarkable findings and for future applications of ethosomes, we will further evaluate the ethosomes’ stability in the bloodstream and evaluate their biodistribution after topical administration in vivo on healthy mouse skin, e.g., by using radio-labeling.

## 5. Conclusions

Our work illustrates that nutlin-3a-loaded ethosomes successfully trigger p53-dependent apoptosis, impede cell migration, and diminish Notch-1 signaling in p53^wild-type^ melanoma cells, indicating a feasible strategy for targeted melanoma treatment. A limitation of this study is related to the absence of in vivo data supporting the efficacy of nutlin-3a-loaded ethosomes against melanoma in the complexity of a living organism. However, the choice of the correct in vivo model is challenging: the ethosomes are not usable for systemic administration but only for topic treatment due to their actual formulation containing 30% ethanol. For this reason, xenograft or PDX mouse models are not appropriate unless visible melanomas are induced on the skin to allow treatment, but to our knowledge, such a model is not yet available [64]. On the other hand, 3D melanoma models, like humanized skin models or 3D culture systems, represent a valuable alternative to animals, closely mimicking tumor complexity compared to 2D cultures. Indeed, models based on different cell types, including vascular and immune components, stratified with melanoma spheroids to mimic the skin tissue, can be useful to assess the effect of nutlin-3a-loaded ethosomes in a tumor-like environment [65].

Furthermore, considering the genetic complexity of melanoma and the relatively high occurrence of drug resistance, it would be interesting to further expand in vitro and in vivo treatments, considering a long-term schedule for the administration of low concentrations of nutlin-3a-loaded ethosomes. Nonetheless, these results and considerations open new perspectives for expanding the use of MDM2 inhibitors in both p53^wild-type^ and p53^mutated^ melanoma. Other future approaches can consider using nutlin-3a in combination with other drugs targeting different pathways to enhance their cytotoxic effects independently of the p53 status of the tumor.

We used melanoma as a proof-of-concept model to assess the effectiveness of the nutlin-3a-loaded ethosomes. Due to the specificity of nutlin-3a, this delivery system has the potential to work as a “nanomedicine” for several skin disorders where p53 can be activated and can exert a biological effect on cells. Examples include, but are not limited to, other cutaneous cancers, like skin squamous cell carcinoma [66], and dermatological diseases, like psoriasis [67].

Even though this is beyond the purpose of the present manuscript, our observations highlight not only an adjuvant role of the ethosomes in the responses to the delivered molecule but also an intrinsic role of the ethosomes in the potential involvement of membrane lipids/lipid rafts and their signaling. For this reason, future studies investigating the effects of ethosomes on lipid rafts could lead to interesting data on melanoma but also on other skin diseases.

## Figures and Tables

**Table 1:** Composition, size distribution parameters, and nutlin-3a entrapment capacity of ethosomes.

Nanodelivery System	Composition (% *w*/*w*)				Z Average ^1^ (nm)	Polydispersity Index	Entrapment Capacity
	Soy Phosphatidylcholine	EtOH	H_2_O	Nutlin-3a			
Ethosomes	0.90	29.10	70.00	-	212.25 ± 14.2	0.12 ± 0.01	-
Nutlin-3a ethosomes	0.90	29.07	70.00	0.03	224.2 ± 11.57	0.17 ± 0.04	89.93 ± 6.70

^1^: As determined by PCS; the data are the mean ± SD of six independent determinations based on different batches.

## Data Availability

Data are contained within this article and the Supplementary Materials.

## Supplementary Materials

The following supporting information can be downloaded at: https://www.mdpi.com/article/10.3390/cells13201678/s1, Figure S1: Nutlin-3a-loaded ethosomes reduce A375 melanoma cell viability; Figure S2: Biocompatibility of ethosomes and nutlin-3a-loaded ethosomes on human keratinocytes; Figure S3: Nutlin-3a-loaded ethosomes induce apoptosis in HT144 melanoma cells.
