# Supplementary material for: Enhanced Anti-Melanoma Activity of Nutlin-3a Delivered via Ethosomes: Targeting p53-Mediated Apoptosis in HT144 Cells
Source: Cells. 2024 Oct 11;13(20):1678. doi: 10.3390/cells13201678 (PMC11506859; doi:10.3390/cells13201678)
Supplement: Supplementary file 1 [file cells-13-01678-s001.zip › cells-3221689-supplementary.pdf]

Supplementary Figures

Enhanced Anti-Melanoma Activity of Nutlin-3a Delivered via Ethosomes: Targeting p53-Mediated Apoptosis in HT144 Cells

Arianna Romani [1,†], Giada Lodi [2,†], Fabio Casciano [2], Arianna Gonelli [3], Paola Secchiero [1], Giorgio Zauli [4], Olga Bortolini [3], Giuseppe Valacchi [3,5,6], Daniele Ragno [7], Agnese Bondi [7], Mascia Benedusi [8], Elisabetta Esposito [7,*] and Rebecca Voltan [2,*]

1 Department of Translational Medicine and LTTA Centre, University of Ferrara, Ferrara 44121, Italy; arianna.romani@unife.it (A.R.); paola.secchiero@unife.it (P.S.)
2 Department of Environmental and Prevention Sciences and LTTA Centre, University of Ferrara, Ferrara 44121, Italy; giada.lodi@unife.it (G.L.); fabio.casciano@unife.it (F.C.)
3 Department of Environmental and Prevention Sciences, University of Ferrara, Ferrara 44121, Italy; arianna.gonelli@unife.it (A.G.); olga.bortolini@unife.it (O.B.); giuseppe.valacchi@unife.it (G.V.)
4 Research Department, King Khaled Eye Specialistic Hospital, Riyadh 12329-8139, Saudi Arabia; gzauli@kkesh.med.sa (G.Z.)
5 Plants for Human Health Institute, Animal Sciences Department, NC Research Campus, NC State University, Kannapolis, NC 28081, USA
6 Department of Food and Nutrition, Kyung Hee University, Seoul 02447, South Korea,
7 Department of Chemical, Pharmaceutical and Agricultural Sciences, University of Ferrara, Ferrara 44121, Italy; daniele.ragno@unife.it (D.R.); agnese.bondi@unife.it (A.B.)
8 Department of Neuroscience and Rehabilitation, University of Ferrara, Ferrara 44121, Italy; mascia.benedusi@unife.it (M.B.)
* Correspondence: elisabetta.esposito@unife.it (E.E.); rebecca.voltan@unife.it (R.V.)
† These authors contributed equally to this work and share first authorship.

SUPPLEMENTARY FIGURES

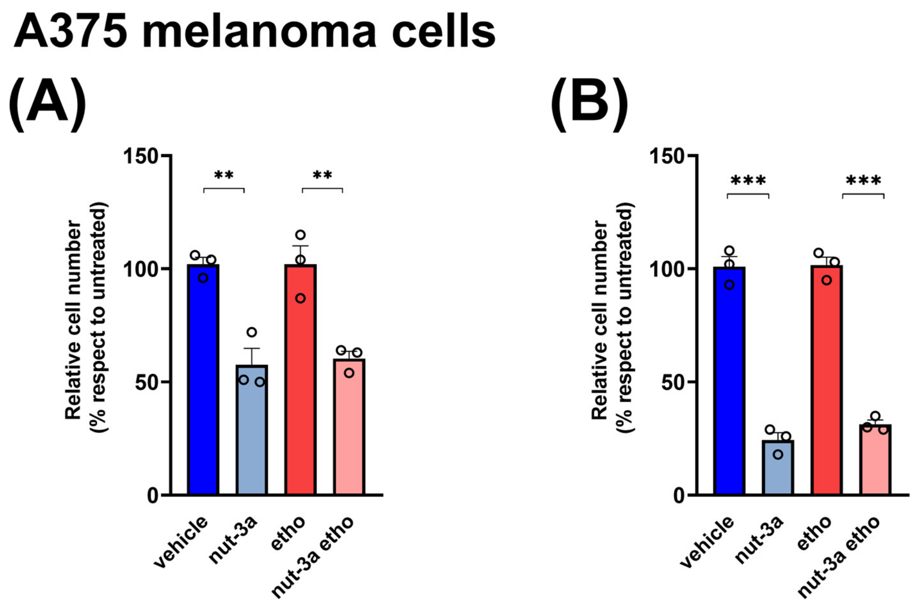

Supplementary Figure S1: Nutlin-3a-loaded ethosomes reduce A375 melanoma cell viability. Cell viability evaluated by Trypan blue dye exclusion of A375 cells, expressing p53 $^{wild\text{-}type}$, treated with 10 µM nutlin-3a (nut-3a) or nutlin-3a loaded ethosomes (nut-3a etho) with equivalent nutlin-3a concentration (10 µM), after 24 (A) and 48 h (B). Vehicle and empty ethosomes (etho) are reported as controls. Data are calculated as percentage with respect to the untreated (set to 100%). Bars represent the mean ± SEM, and each circle denotes the value of each experimental replicate. Statistical analysis was performed by ANOVA, followed by Bonferroni's post hoc test. The number of asterisks indicates the relative p-value *(p)*: **$p \leq 0.01$, ***$p \leq 0.001$.

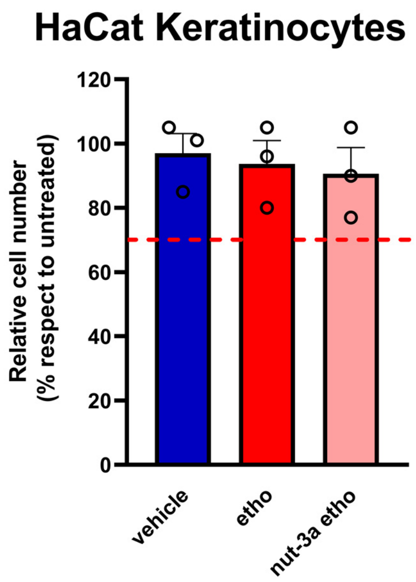

Supplementary Figure S2: Biocompatibility of ethosomes and nutlin-3a-loaded ethosomes on human keratinocytes. Cell viability evaluated by Trypan blue dye exclusion of HaCat keratinocytes cell culture treated with nutlin-3a loaded ethosomes (nut-3a etho) with nutlin-3a concentration of 10 µM, after 24 h. Vehicle and empty ethosomes (etho) are reported as controls. Data are calculated as percentage with respect to the untreated (set to 100%) and reported as mean ± standard error of the mean. Bars represent the mean ± SEM, and each circle denotes the value of each experimental replicate. Statistical analysis was performed by ANOVA, followed by Bonferroni's post hoc test. No significant differences were reported. Red dotted line represents the threshold for cytotoxicity settled at 70% of viability.

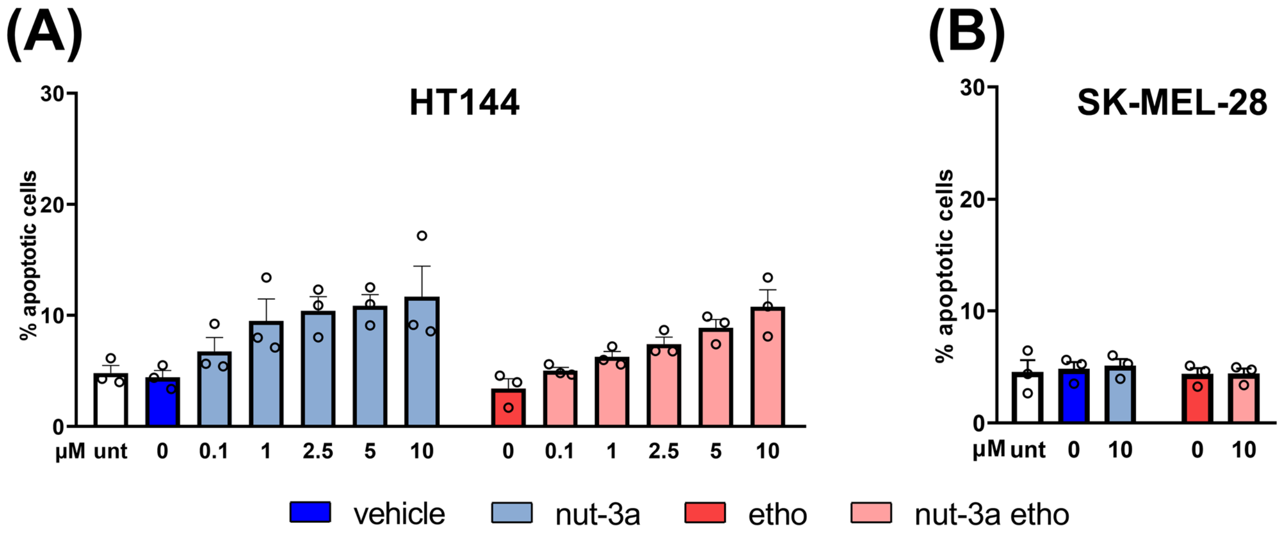

Supplementary Figure S3: Nutlin-3a-loaded ethosomes induce apoptosis in HT144 melanoma cells. Apoptosis level of HT144 expressing p53$^{wild\text{-}type}$ (A) and SK-MEL-28 expressing p53^{mut} (B) cultures treated with nutlin-3a or nutlin-3-loaded ethosomes with equivalent nutlin-3a concentrations, after 24 h. Vehicle and empty ethosomes are reported as controls. Results are expressed as a percentage of the total population. Bars represent the mean ± SEM, and each circle denotes the value of each experimental replicate. Statistical analysis was performed by ANOVA. No significant differences were reported.
